# Sulfiredoxin stimulates luteinization *in vitro* by promoting progesterone production in rats

**DOI:** 10.1530/REP-25-0066

**Published:** 2025-09-19

**Authors:** Sang-Young Chun, Eun-Hye Park, Mari Jang, Jae-Il Park, You-Jee Jang

**Affiliations:** ^1^ School of Biological Sciences & Technology, Faculty of Life Science, Chonnam National University, Gwangju, Republic of Korea; ^2^ Department of Biomedical Laboratory Science, Honam University, Gwangju, Republic of Korea; ^3^ Korea Basic Science Institute, Honam Regional Center, Gwangju, Republic of Korea

**Keywords:** sulfiredoxin (Srxn1), corpus luteum, ovulation, luteinization, J14 inhibitor

## Abstract

**In brief:**

Sulfiredoxin (Srxn1) is essential for corpus luteum formation during ovulation. Inhibition of Srxn1 with J14 suppressed LH-stimulated progesterone production, key gene expressions (*Cyp11a1*, *Star*), and markers of luteinization. This highlights Srxn1’s role in promoting LH-induced luteinization through the ERK, C/EBPβ, and Cyp11a1 pathways.

**Abstract:**

Sulfiredoxin (Srxn1), an antioxidant enzyme, is expressed during ovulation. This study aimed to investigate the physiological function of Srxn1 in corpus luteum formation during the ovulatory process in rats. Treatment of cultured preovulatory follicles with the Srxn1 inhibitor, J14, suppressed LH-stimulated progesterone production, but not estradiol production, in a dose-dependent manner. Likewise, LH-stimulated gene expression of *Cyp11a1* and *St*ar, but not *Cyp19a1*, was suppressed by J14. Regulation of Cyp11a1 and STAR expression by Srxn1 was confirmed using si-Srxn1 in KGN cells, a human granulosa cell line. Furthermore, treatment of preovulatory granulosa cells with J14 dose-dependently suppressed LH-stimulated mRNA and protein levels of C/EBP*β* and ERK1/2. Finally, hypertrophy, lipid droplets, progesterone production, and p27Kip1 expression stimulated by LH were significantly lowered by J14 in *in vitro* luteinization of granulosa cells. Taken together, the present data indicate that Srxn1 plays a significant role in the formation of the corpus luteum during ovulation by stimulating the pathways of ERK, C/EBP*β*, and *Cyp11a1*.

## Introduction

Sulfiredoxin 1 (Srxn1) was initially identified as an antioxidant enzyme that regulates reactive oxygen species (ROS) signaling by reducing the inactive hyperoxidized 2-cysteine peroxiredoxins (2-Cys Prdxs; Prdx 1–4) (Biteau *et al.* 2003, Woo *et al.* 2003). Numerous studies have shown that inactive 2-Cys Prdxs are selectively reduced by Srxn1 to exert its physiological functions (Jeong *et al.* 2006, Perkins *et al.* 2014, Ramesh *et al.* 2014). Steroidogenic tissue-specific ablation of Srxn1 results in the accumulation of inactive hyperoxidized Prdx3, thereby suppressing adrenal corticosterone production in mice (Kil *et al.* 2012). Alternative enzymatic functions of Srxn1 include catalyzing the deglutathionylation of protein tyrosine phosphatase 1B (PTP1B) and actin (Findlay *et al.* 2006). As a central endogenous antioxidant protein, Srxn1 plays an important role in various physiological processes including cell apoptosis, invasion, and tumorigenesis by regulating oxidative stress-triggered cell damage (Bell *et al.* 2011, Ramesh *et al.* 2014, Zhou *et al.* 2015). It is ubiquitously expressed in mammalian tissues, and its expression is regulated by various factors, including endogenous hormones such as adrenocorticotropic hormone in the adrenal cortex of mice and luteinizing hormone (LH) in rat ovaries (Ramesh *et al.* 2014).

Gonadotropins transiently induce Srxn1 during ovulation in rats. Reactive OS play an important role in ovulation in rodents (Shkolnik *et al.* 2011, Park *et al.* 2012). Prdx1 and Prdx2 accumulate in preovulatory follicles (Park *et al.* 2012) and regulate cumulus expansion during ovulation, as shown in Prdx1-deficient (Park *et al.* 2021) and Prdx2-deficient mice (Jang *et al.* 2020), respectively. Although its expression has been reported, the specific biological functions of Srxn1 during ovulation remain unclear.

The purpose of the present study was to investigate the role of Srxn1 in luteinization in preovulatory granulosa cells of rats. Our findings demonstrate that Srxn1 enhances progesterone production by activating the extracellular signal-regulated kinase (ERK)-1/2 and CCAAT/enhancer-binding protein (C/EBP)-β pathway, thereby promoting luteinization *in vitro*.

## Materials and methods

### Hormones and reagents

Ovine LH was obtained from the National Hormone and Pituitary Distribution Program (NIDDK, USA). Sulfiredoxin inhibitor, J14, was purchased from AMRI (India) and prepared as reported previously (Kim *et al.* 2016). Antibodies against C/EBPβ, p44/42 MAPK (ERK1/2), phospho-p44/42 MAPK, and p27Kip1 were purchased from Cell Signaling Technology, Inc. (USA). An antibody against glyceraldehyde-3-phosphate dehydrogenase (GAPDH) was obtained from Santa Cruz Biotechnology (USA). Forskolin, PMA (phorbol 12-myristate 13-acetate), and H-89 (A-kinase inhibitor) were purchased from Sigma (USA).

### Animals

Immature female Sprague-Dawley rats (Samtako BioKorea, Korea) were housed in groups in a temperature-controlled room under a 14 h light:10 h darkness cycle (lights on from 06:00 to 20:00 h). The rats had free access to food and water. At 26 days of age, rats (body weight, 60–65 g) were injected subcutaneously with 10 IU of eCG (Sigma) to induce multiple follicle growth. Two days later, rats were euthanized to collect preovulatory follicles and granulosa cells for *in vitro* culture. All rats were maintained and treated in accordance with the National Institutes of Health Guidelines for the Care and Use of Experimental Animals, as approved by the Institutional Animal Care and Use Committee at Chonnam National University and Korea Basic Science Institute (KBSI-AEC 1516).

### Culture of preovulatory follicles and granulosa cells

To study steroidogenesis, preovulatory follicles were isolated from ovaries collected 48 h after eCG injection, and follicle culture was performed as previously described (Park *et al.* 2001). Five follicles were cultured in glass vials containing 300 μL αMEM (Life Technologies, Inc., USA) supplemented with penicillin, streptomycin, L-glutamine, and 0.1% BSA at 37°С under 5% CO_2_–95% O_2_. The incubation was maintained for 24 h without changing the culture media. Sulfiredoxin inhibitor, J14 (30 μM), was added 30 min before treatment with LH (200 ng/mL). At the end of incubation, the media were snap-frozen to conduct an enzyme-linked immunosorbent assay (ELISA) for the measurement of steroid hormones.

To isolate granulosa cells from preovulatory follicles, ovaries were flattened to one layer to easily identify preovulatory follicles in phosphate-buffered saline, and granulosa cells were obtained by the follicular puncture method using a 23-gauge needle under a dissection microscope. Cells were cultured at a density of 1 × 10^6^ cells per tube (Falcon 2063; Becton Dickinson Labware, USA) in DMEM/Ham’s F-12 supplemented with antibiotics and 0.1% BSA at 37°C in a humidified atmosphere of 5% CO_2_. The incubation was maintained for 24 h without changing the culture media. Sulfiredoxin inhibitor, J14 (30 μM), was added 30 min before treatment with LH (200 ng/mL). All biological samples including follicles and granulosa cells were kept on ice during the isolation and preparation process. At the end of the culture, the cells were snap-frozen to measure mRNA and protein levels.

### Enzyme-linked immunosorbent assay for the measurement of steroid hormones

Levels of progesterone and estradiol in the medium were assayed using an enzyme-linked immunosorbent assay (ELISA) (Arbor Assays, USA) with detection limits ranging from 50 pg/mL to 3,200 pg/mL. Samples were diluted either 1:20 or 1:50 with assay buffer, and all procedures were performed according to the manufacturer’s instructions.

### RNA isolation and real-time PCR analysis

Total RNA was extracted from the preovulatory follicles and granulosa cells using Hybrid-R^TM^ (GeneAll, Korea) according to the manufacturer’s instructions. Whole follicles or isolated granulosa cells were homogenized in lysis buffer for RNA extraction as previously described (Park *et al.* 2012). Total RNA was then reverse-transcribed with the M-MLV cDNA synthesis kit (Enzynomics, Korea) to evaluate gene expression. RT-PCR was performed using the Rotor-Gene 3000 thermocycler (Corbett Research, Australia) with the QuantiTect SYBR Green PCR kit (QIAGEN, Germany). Primers were designed using PRIMER3 software and are listed in Supplemental Table S1 (see section on Supplementary materials given at the end of the article). The average Ct value in triplicate for each gene was normalized to the linear C value of β-actin to obtain the abundance of transcript.

### Western blot analysis

Granulosa cell lysates (50 μg protein per lane) were resolved by 8% SDS-polyacrylamide gel electrophoresis and transferred to nitrocellulose membranes (Amersham Bioscience, USA). After blocking with 3% skim milk, the membranes were incubated with a primary antibody (1:500 final dilution), followed by a horseradish peroxidase-conjugated secondary IgG (1:1,000 final dilution). After washing with 1 × Tris-buffered saline with Tween-20 (TBST), reactive bands were visualized with enhanced chemiluminescence (Amersham). Band intensities were quantified using UN-SCAN-IT Gel 6.1 software (Silk Scientific, USA) after subtracting background signals. GAPDH was used as a loading control.

### Small interfering RNA (siRNA) transfection

The human granulosa cell tumor-derived cell line KGN was kindly provided by Dr Toshihiko Yanase at Fukuoka University (Fukuoka, Japan). Cells (1 × 10^5^) were cultured in DMEM/F-12 containing 10% fetal bovine serum (FBS) without antibiotics in 6-well plates in a humidified atmosphere with 5% CO_2_ and 95% air at 37°C. A specific small interfering RNA (siRNA) targeting human Srxn1 as well as a scrambled control siRNA (Santa Cruz, TX, USA) was transfected into KGN cells via electroporation using the Neon Transfection System (Invitrogen, USA) according to the manufacturer’s instructions. Four hours after transfection, the cells were incubated for 8 h in serum-free fresh DMEM/F-12 containing forskolin (1 μM) and PMA (20 nM) to mimic the LH action, with or without a pharmacological inhibitor, J14 (30 μM). After incubation, cellular RNA was extracted and subjected to real-time PCR to quantify the expression of Cyp11a1, Star, and Srxn1.

### Luteinization *in vitro*

The procedures for luteinization of granulosa cells *in vitro* were conducted as reported previously (Morris & Richards 1995). Preovulatory follicles were dissected from ovaries of immature rats primed with eCG for 48 h. Follicles (∼30 follicles/vial) were incubated in serum-free conditions for 7 h in 1 mL of DMEM/F-12 at 37°C with 95% oxygen-5% CO_2_ in the presence of LH (200 ng/mL), with or without H-89 (A-kinase inhibitor, 10 μM) or J14 (Srxn1 inhibitor, 30 μM). After incubation, granulosa cells were collected from the follicles for each treatment group (four vials per treatment) by puncturing with forceps, washed once in DMEM/F-12, resuspended in DMEM/F-12 containing 1% FBS, and then plated into the central glass-bottom Tomodish (Tomocube, Korea). The cells were cultured for 10 days with a change of medium every 3 days until assayed.

On day 10 of culture, medium samples were collected, boiled for 3 min, and stored at −20°C for progesterone assay. Photomicrographs of a luteal cell phenotype, including nuclear hypertrophy and increased lipid droplets, were also taken using Bodipy 493/503 (green).

### Statistical analysis

Data are presented as means ± SEM. Comparisons between any two points were evaluated using Student’s two-tailed *t*-test. One-way analysis of variance (ANOVA), followed by Dunnett’s test, was used for comparisons among multiple groups. *P* < 0.05 was considered significant.

## Results

### Srxn1 regulates progesterone production in the cultured preovulatory granulosa cells

**Figure 1:**
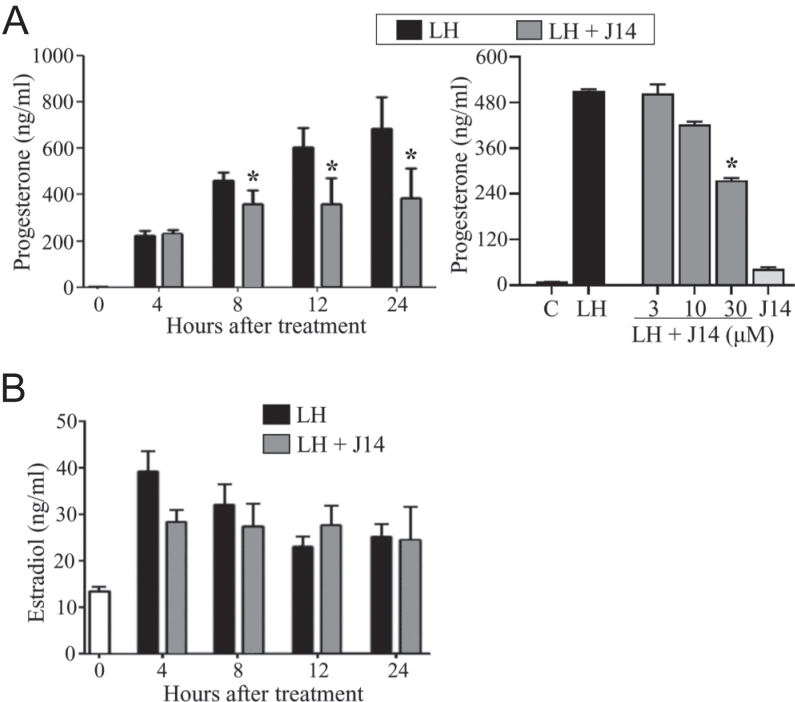
Effect of Srxn1 on steroid production in cultured preovulatory follicles. Preovulatory follicles (5 follicles/tube) obtained from ovaries of immature rats injected with eCG for 48 h were cultured in serum-free medium in the absence (C; control) or presence of LH (200 ng/mL), with or without J14 (30 μM maximum), an Srxn1 inhibitor. Concentrations of progesterone (A) and estradiol (B) in the medium were determined using ELISA. Note that the dose-dependent effect of J14 on progesterone production was determined at 12 h of culture. Data are the mean ± SEM of three independent experiments. *P < 0.05 vs corresponding LH treatment*.*

To examine the effect of Srxn1 on steroidogenesis, the concentrations of progesterone and estradiol were measured in media of cultured preovulatory follicles after LH treatment, with or without the Srxn1 inhibitor, J14 (Fig. 1). Progesterone production gradually increased until 24 h after LH treatment (Fig. 1A). However, J14 suppressed this effect from 8 to 24 h of culture (*P* < 0.05). In addition, it suppressed LH-stimulated progesterone production in a dose-dependent manner at 12 h of culture. Notably, J14 did not alter the LH-stimulated estradiol levels during 24 h of culture (Fig. 1B).

**Figure 2:**
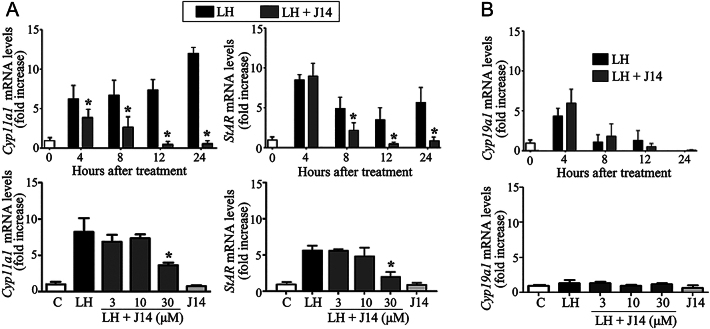
Effect of Srxn1 on levels of transcripts encoding steroidogenic enzymes in cultured granulosa cells of preovulatory follicles. Granulosa cells of preovulatory follicles were cultured in serum-free medium in the absence (C; control) or presence of LH (200 ng/mL), with or without an Srxn1 inhibitor, J14 (3–30 μM). The mRNA levels of *Cyp11a1* and *Star* (A), and *Cyp19a1* (B) were measured by real-time PCR. Note that the dose-dependent effect of J14 on steroid production was determined at 8 h of culture. The expression of the target transcript was normalized against that of β-actin. Data are the mean ± SEM of three independent experiments. *P < 0.05 vs corresponding LH treatment*.*

Next, the effects of Srxn1 on steroidogenic enzyme expression levels were examined using cultured preovulatory granulosa cells. Cytochrome P450 family 11 subfamily A member 1 (Cyp11a1) and steroidogenic acute regulatory protein (*Star*) mRNA levels increased after LH treatment until 24 h of culture (Fig. 2A). Exposure of granulosa cells to 30 μM J14 inhibited the LH-stimulated Cyp11a1 expression from 4 to 24 h of culture and Star expression from 8 to 24 h of culture. Notably, exposure to 30 μM J14, but not 3 and 10 μM J14, for 8 h inhibited both Cyp11a1 and Star expression in the granulosa cells (*P* < 0.05). Treatment with 30 μM J14 alone did not affect the enzyme expression levels. Moreover, exposure of granulosa cells to J14 did not affect the LH-stimulated *Cyp19a1* mRNA levels during 24 h of culture (Fig. 2B).

**Figure 3:**
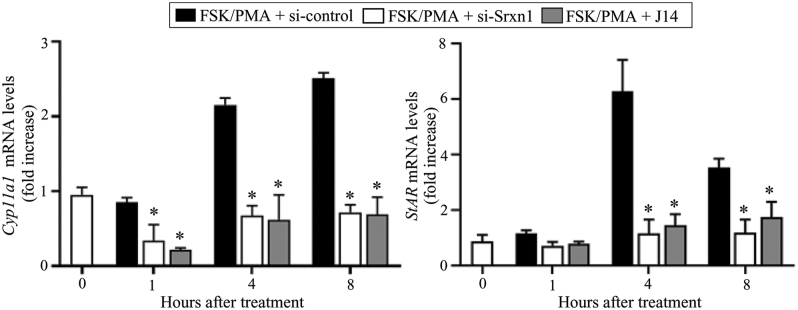
Effect of Srxn1 on levels of transcripts encoding steroidogenic enzymes in human granulosa-like KGN cells. KGN cells (1 × 10^5^ cells/mL) were transfected with 1 M control siRNA (si-control) or Srxn1 siRNA (si-Srxn1) for 48 h, and then treated with forskolin (FSK; 1 μM) and PMA (20 nM) for up to 8 h. Some cells were also treated with the Srxn1 inhibitor, J14 (30 μM). The mRNA levels of *Cyp11a1* and *Star* were examined by real-time PCR. The expression of the target transcript was normalized against that of β-actin. Data are the mean ± SEM of three independent experiments. *P < 0.05 vs corresponding FSK/PMA treatment*.*

The stimulatory effect of Srxn1 on the expression of steroidogenic enzymes was confirmed using small interfering Srxn1 RNA (si-Srxn1) in human granulosa-like KGN cells. Treatment with forskolin and phorbol 12-myristate 13-acetate (PMA) (FSK/PMA), which mimic the LH action, stimulated the gene expression of *Cyp11a1* and *Star* during 4–8 h of culture (Fig. 3) as well as Srxn1 (Supplemental Fig. 1).

### Srxn1 activates the C/EBPβ and ERK1/2 signaling pathways in the preovulatory granulosa cells

**Figure 4:**
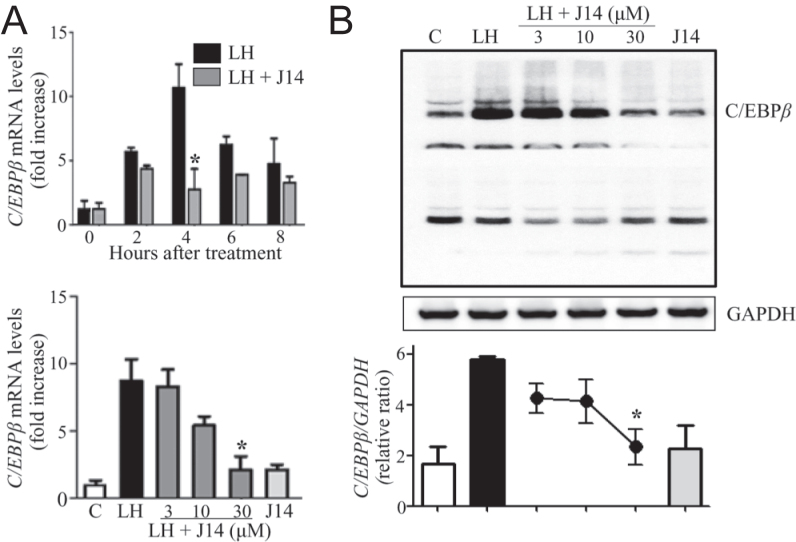
Effect of Srxn1 on the expression of C/EBPβ in cultured granulosa cells of preovulatory follicles. Granulosa cells of preovulatory follicles were cultured in serum-free medium in the absence (C; control) or presence of LH (200 ng/mL), with or without an Srxn1 inhibitor, J14 (3–30 μM). The mRNA (A) and protein (B) levels of C/EBPβ were measured by real-time PCR and Western blot analysis, respectively. The mRNA levels are expressed as relative amounts of GAPDH. The protein levels were quantified using a phosphorimager and normalized to GAPDH levels. Data are the mean ± SEM of three independent experiments. *P < 0.05 vs corresponding LH treatment*.*

**Figure 5:**
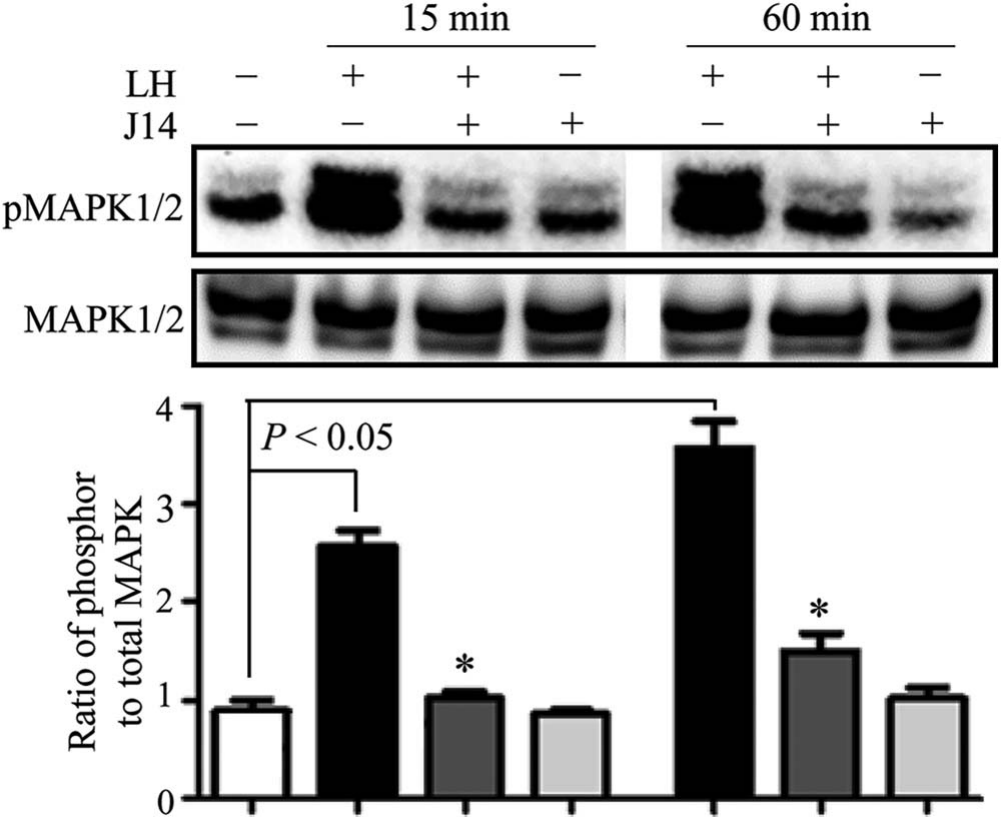
Effect of Srxn1 on the activation of MAPK1/2 in cultured granulosa cells of preovulatory follicles. Granulosa cells of preovulatory follicles were cultured in serum-free medium in the absence or presence of LH (200 ng/mL), with or without an Srxn1 inhibitor, J14 (30 μM) for 15 and 60 min. MAPK1/2 activity was detected in lysates (50 μg/lane) by immunoblotting using anti-pMAPK1/2 antibody. The protein levels were quantified using a phosphorimager and normalized to internal standard MAPK1/2 levels. Data are the mean ± SEM of three independent experiments. *P < 0.05 vs corresponding LH treatment*.*

As C/EBPβ regulates both Cyp11a1 and Star expression levels (Fan *et al.* 2011), the effect of J14 on the expression of *C/ebpβ* was examined in cultured granulosa cells. Levels of *C/ebpβ* mRNA increased within 2 h, were highest at 4 h, and remained present at 8 h after LH treatment (Fig. 4A). However, 30 μM J14 significantly suppressed the LH-stimulated expression of the *C/ebpβ* gene at 4 h (*P* < 0.05). Moreover, J14 suppressed LH-stimulated *C/ebpβ* expression in a dose-dependent manner, showing highest suppression at 30 μM. Similarly, C/EBPβ protein expression was stimulated by LH after 4 h of treatment; however, the addition of J14 dose-dependently suppressed this effect (Fig. 4B). Notably, J14 alone did not affect the C/EBPβ mRNA and protein levels. As C/EBPβ is a downstream effector of ERK1/2 during ovulation (Fan *et al.* 2009), the effect of the Srxn1 inhibitor on mitogen-activated protein kinase (MAPK)-1/2 activation was examined in the cultured granulosa cells. LH activated MAPK1/2 by 2.6-fold after 15 min and 3.4-fold after 60 min of treatment (Fig. 5). However, 30 μM J14 significantly inhibited the LH-stimulated MAPK1/2 activity at both 15 and 60 min. Treatment with J14 alone did not affect the MAPK1/2 activity.

### Srxn1 induces the differentiation of preovulatory granulosa cells into luteal cells

**Figure 6:**
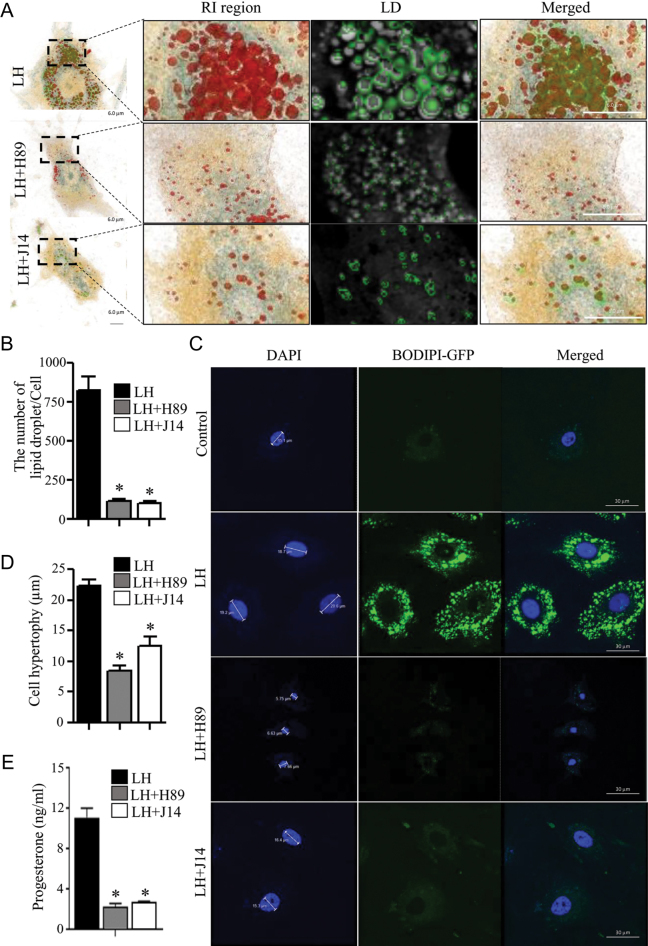
Effect of Srxn1 on luteinization *in vitro*. Preovulatory follicles were cultured for 7 h in the presence of LH (500 ng/mL), with or without H89 (10 μM, PKA inhibitor) or J14 (30 μM, Srxn1 inhibitor). Granulosa cells were then harvested from follicles and cultured in DMEM-F12 containing 1% FBS for 10 days, with the change of media every 3 days. To demonstrate the morphological features of corpus luteum formation, photographs were taken on day 10 after staining with Bodipy for lipid droplets (A) using a holotomography microscope, and after staining for nuclear hypertrophy (C) using a fluorescence microscope. (B) Quantitation of the number of lipid droplets from photomicrographs A. (D) Quantitation of cell hypertrophy size from photomicrographs C. (E) Progesterone concentrations in media are the mean ± SEM of three different samples. *P < 0.05 vs corresponding LH treatment.

C/EBPβ mediates the terminal differentiation of granulosa cells during luteinization by regulating steroidogenic enzymes such as Cyp11a1 and Star (Fan *et al.* 2011). To determine the role of Srxn1 in corpus luteum formation, we employed an *in vitro* luteinization system as previously described (Morris & Richards 1995). Granulosa cells obtained from the preovulatory follicles exposed to LH for 7 h and cultured for 10 days in medium containing 1% FBS displayed prominent nuclei and exhibited the luteal cell phenotype, i.e. increased lipid droplets and cell hypertrophy (Fig. 6A and C), as observed by holotomography and fluorescence microscopy, respectively. In contrast, luteinized granulosa cells treated with 10 μM H-89, a PKA (A-kinase) inhibitor, showed an 80% reduction in the number of lipid droplets (Fig. 6B) and a 55% reduction in cell hypertrophy (Fig. 6D). Interestingly, cells harvested from follicles exposed to both LH and 30 μM J14 also showed inhibition of LH-induced luteinization, with a 50% reduction in nuclear hypertrophy and an 80% reduction in the number of lipid droplets (*P* < 0.05). After 10 days of granulosa cell culture, in addition to changes in phenotype, progesterone production in the medium was significantly reduced when the cells were harvested from follicles incubated with LH and either H-89 or J14, compared to cells harvested from follicles incubated with LH alone (Fig. 6E).

**Figure 7:**
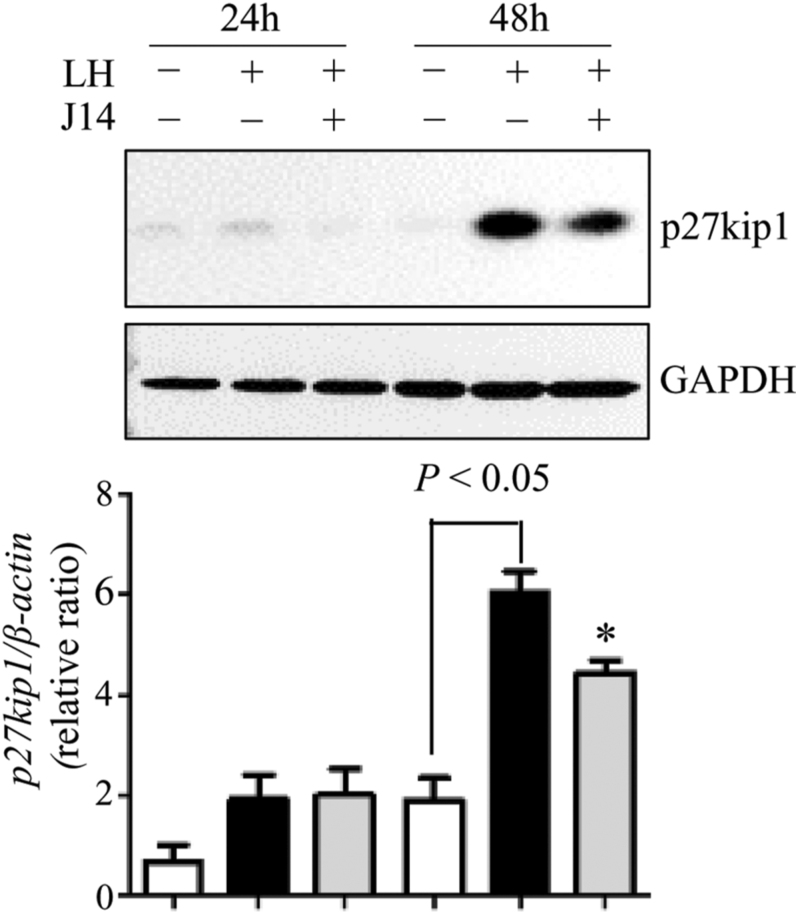
Effect of Srxn1 on p27Kip1 expression in cultured granulosa cells of preovulatory follicles. Granulosa cells of preovulatory follicles were cultured in serum-free medium in the absence or presence of LH (200 ng/mL), with or without an Srxn1 inhibitor, J14 (30 μM), for 24 and 48 h. The p27Kip1 expression was measured by Western blot. The protein levels were quantified using a phosphorimager and normalized to GAPDH levels. Data are the mean ± SEM of three independent experiments. *P < 0.05 vs corresponding LH treatment*.*

Luteinization is characterized by the cessation of cell proliferation, primarily mediated by increased expression of the cell cycle inhibitor p27Kip1, which regulates cell cycle exit and promotes terminal differentiation of granulosa cells (Rajareddy *et al.* 2007). To assess the effect of Srxn1 on p27Kip1 expression, lysates of cultured granulosa cells were used for Western blot analysis. Cells treated with LH for 48 h, but not 24 h, exhibited a significant increase in p27Kip1 expression (Fig. 7). Interestingly, the addition of 30 μM J14 reduced LH-stimulated p27Kip1 expression at 48 h culture (*P* < 0.05).

## Discussion

Srxn1 expression is stimulated during ovulation (Park *et al.* 2012); however, its specific physiological roles remain unclear. In this study, we found that Srxn1 played a critical role in corpus luteum formation *in vitro* by enhancing progesterone production via the ERK1/2–C/EBPβ pathway. This finding is consistent with previous reports showing decreased progesterone production in ERK1/2 (Fan *et al.* 2009) and C/EBPβ (Fan *et al.* 2011) knockout mice. LH-induced luteal cell phenotypes *in vitro*, including morphological hypertrophy and increased progesterone production (Morris & Richards 1995), were largely disrupted by the Srxn1 inhibitor. These results suggest that LH-induced Srxn1 expression during ovulation promotes luteinization by enhancing progesterone production. In addition to Srxn1, ROS (Shkolnik *et al.* 2011), Prdx1 (Park *et al.* 2021), and Prdx2 (Jang *et al.* 2020) are involved in the ovulatory process in rodents. Srxn1 may promote luteinization by regulating intracellular ROS levels via the restoration of peroxiredoxins (Prdxs), which in turn influence progesterone production. Reactive OS appear to have dual roles; high levels of ROS act as an oxidative stressor, and moderate levels of ROS act as a signal molecule (Rhee 2006). Excessive ROS during ovulation can impair luteal function by disrupting LH receptor signaling and modulating progesterone production (Nose *et al.* 1991, Kato *et al.* 1997, Sugino 2006). In contrast, LH induced a rapid increase in ROS production within 15 min in cultured preovulatory follicles, which subsequently upregulates Srxn1 expression, indicating that ROS function as signaling molecules (Park *et al.* 2012). Srnx1 regulates H_2_O_2_-mediated signaling by restoring hyperoxidized 2-Cys Prdxs, thereby modulating cell proliferation and survival through control of cell cycle regulators such as p21 and p27, suggesting a potential role in granulosa cell function and differentiation during ovulation (Robker & Richards 1998). In the present study, LH-induced expression of p27 was significantly reduced by treatment with the Srx inhibitor, J14, indicating that Srxn1 is critical not only for progesterone production but also for maintaining ROS balance in preovulatory follicles during ovulation and luteinization.

Antioxidant mechanisms are essential for the maintenance of the corpus luteum and its function in progesterone production, which is crucial for implantation and early embryonic development (Al-Gubory *et al.* 2012). Srxn1 restores Prdx to its active state, thereby regulating intracellular ROS levels (Biteau *et al.* 2003). LH induces the production of ROS, which are vital preovulatory signaling molecules. These ROS facilitate the activation of the epidermal growth factor (EGF) receptor and p42/44 mitogen-activated protein kinase (MAPK) signaling, ultimately promoting progesterone synthesis (Terasaka *et al.* 2017). Srxn1 may enhance progesterone production via sequential activation of ERK1/2, C/EBPβ, and Cyp11a1/StAR. Furthermore, Srxn1 catalyzes the deglutathionylation of PTP1B, restoring its phosphatase activity (Cho *et al.* 2004, Findlay *et al.* 2006), which inhibits the MAPK1/2 pathway. However, in this study, inhibition of LH-induced MAPK1/2 activity by the Srxn1 inhibitor confirmed that Srxn1 positively regulates ERK1/2, suggesting it may modulate this pathway through additional mechanisms beyond PTP1B regulation. Indeed, the PKA pathway plays a role in the activation of ERK1/2 in granulosa cells during the ovulatory process (Fan *et al.* 2011). The Srxn1 inhibitor also suppressed LH-induced C/EBPβ expression, indicating the activation of C/EBPβ by ERK1/2. A previous study on C/EBPβ knockout mice has demonstrated the essential roles of C/EBPβ induced by ERK1/2 signaling in regulating follicle rupture, corpus luteum formation, and steroidogenesis in granulosa cells (Fan *et al.* 2011). C/EBPβ increases progesterone production via transcriptional activation of Cyp11a1 and StAR (Fan *et al.* 2011). In this study, both Cyp11a1 and Star expression levels were reduced by the Srxn1 inhibitor and si-Srxn1, suggesting the cascade activation of ERK1/2, C/EBPβ, and Cyp11a1/StAR by Srxn1.

Srxn1-mediated ERK1/2 activation may also induce epigenetic modifications such as histone trimethylation in the Cyp11a1 and Star promoter. Specifically, ERK1/2-induced trimethylation of histone H3 lysine 4 (H3K4me3) has been linked to activation of these genes (Maekawa *et al.* 2016). By regulating ROS levels, Srxn1 possibly influences these epigenetic processes, affecting histone modifications to maintain cellular redox homeostasis during ovulation. In addition, epigenetic regulation of Prdx genes provides another layer of control over ROS balance (Dutta *et al.* 2024). Srxn1 is also known to play a role in cellular stress responses, including cell survival and apoptosis (Li *et al.* 2018). As the EGF-R pathway influences granulosa cell physiology during ovulation, changes in Srxn1 expression may be associated with EGF-R signaling activation (Conti *et al.* 2006). In addition to restoring inactive Prdx activity, Srxn1 possibly performs other independent actions during ovulation. Reactive OS plays a role in ovulation (Shkolnik *et al.* 2011), and Prdx1 (Park *et al.* 2021) and Prdx2 (Jang *et al.* 2020) contribute to the expansion of the cumulus–oocyte complex. However, treatment of preovulatory granulosa cells or the cumulus–oocyte complex with the Srxn1 inhibitor did not affect cumulus expansion or oocyte maturation in this study (data not shown), suggesting that Srxn1 functions independently of the ROS–Prdx pathway.

Studies using fully differentiated granulosa cells from preovulatory follicles have been limited due to low transfection efficiency and the loss of key features such as LH receptors under standard culture conditions (Sriraman *et al.* 2003). Furthermore, no established cell lines fully replicate the phenotype of mature follicular granulosa cells. Therefore, the direct physiological role of Srxn1 could thus not be tested using a knockdown approach in the cellular model of the ovulatory granulosa cells (Havelock *et al.* 2004). We therefore used the Srxn1 inhibitor J14 in primary cultures of preovulatory granulosa cells to study its function. In addition, one of the limitations of the present study is the lack of a control compound for the Srxn1 inhibitor J14. A control compound such as an inactive chemical form of J14 is not commercially available. To circumvent this limitation, the present study used specific doses of the Srxn1 inhibitor without toxic effect based on a previous report on the selective induction of cancer cell death by J14 (Kim *et al.* 2016).

Our findings show that Srxn1 inhibition suppressed progesterone but not estrogen production, suggesting that Srxn1 is involved in the LH-induced shift from an estrogen-dominant to a progesterone-dominant environment during ovulation. The LH surge stimulates Cyp11a1 and Star expression while downregulating aromatase, thereby facilitating this hormonal shift (Richards *et al.* 2002). Estrogen is known to reduce oxidative stress, whereas progesterone tends to increase it, indicating a complex interaction between steroid hormones and oxidative balance in reproductive physiology (Cagnacci *et al.* 2023).

In summary, Srxn1 contributes to luteinization during the ovulatory process by activating the ERK1/2 signaling pathway. Further studies are needed to clarify the physiological function of Srxn1 during ovulation by generating Srxn1 knockout mice in granulosa cells of preovulatory follicles. Understanding the function of Srxn1 could pave the way for novel therapeutic strategies to treat disorders related to oxidative stress, such as female infertility associated with luteal phase defects.

## Supplementary materials



## Declaration of interest

The authors declare that there is no conflict of interest that could be perceived as prejudicing the impartiality of the work reported.

## Funding

This work was supported by Honam Universityhttps://doi.org/10.13039/501100002495 grant (2022-0141 to Y-JJ), and Gwangju Regional Innovation System & Education (RISE) Project (2025-0107 to Y-JJ), and Korea Basic Science Institutehttps://doi.org/10.13039/501100003716 grant (C523400 to J-IP).

## Author contribution statement

S-YC and Y-JJ designed the study. J-IP, E-HP, MJ, and Y-JJ conducted the experiments and analyzed the data. S-YC and Y-JJ were responsible for writing, reviewing, and editing. All other authors reviewed, revised, and approved the final manuscript.
