# Supplementary material for: Sulfiredoxin stimulates luteinization in vitro by promoting progesterone production in rats
Source: Reproduction. 2025 Sep 19;170(4):e250066. doi: 10.1530/REP-25-0066 (PMC12449702; doi:10.1530/REP-25-0066)
Supplement: Supplementary file 1 [file supplementary_materials.pdf]

Supplemental Table 1. Primers used for determination of mRNA levels by RT-PCR

Gene	Rat		Human	
	Primer sequence (5'→3')	Size (bp)	Primer sequence (5'→3')	Size (bp)
Cyp11a1	F: TCACATGCAGAATTTCCAGAAG<br>B: AGGATGTAACTGACTCCATGTTG	530-911 (381)	F: CAATGAGATCCCCTCTCCTG<br>B: GCCTTTGAGTCCATCACTAAC	188-680 (563)
StAR	F: GATTGGAAAAGACACAGTCATCACC<br>B: GTGAGTTTGGTCTTTGAGGGACTTC	593-836 (243)	F: GGACAAAGTGATGAGTAAAGT<br>B: ACATGCCTGAGCAGAAGGG	449-775 (327)
Cyp19a1	F: GCTTCTCATCGCAGAGTATCCGG<br>B: CAAGGGTAAATTCATTGGGCTTGG	1,569-1859 (290)	-	
C/EBPβ	F: ATCGACTTCAGCCCCTACCT<br>B: CGACTACGGTTACGTGAGCC	234-434 (200)	-	
Srxn1	-		F: CCCATCGATGTCCTCTGGATCAAA<br>B: AGGTACACCCTTAGGTCTGAGAGA	246-401 (155)
β-actin	F: GAGACCTTCAACACCCCAGCC<br>B: CCGTCAGGCAGCTCATAGCTC	373-734 (362)	F: GAGACCTTCAACACCCCAGCC<br>B: CCGTCAGGCAGCTCGTAGCTC	456-818 (323)
